# Alveolar Abscess

**Published:** 1868-10

**Authors:** W. H. Shadoan


### ALVEOLAR ABSCESS.

BY DR. W. H. SHADOAN.

[Continued from page 363.]

### IODINE AND ITS TINCTURES.

Iodine is an elementary non-metalic substance having some resemblance to chlorine. It was discovered in 1812, by a soda manufacturer of Paris. Sometime after this its therapeutic properties were discovered, since which, it has gradually come into general use, so that at the present time it is universally a standard remedy. It is found chiefly to exist in the kelp of sea weeds, in the animal, and mineral kingdom. It is also found as an iodide of sodium in several mineral springs of the United States, and in some minerals in other parts of the country. As a therapeutic agent, iodine is used as an absorbent—it excites absorption in the alveolus, and in erysipelatious affections. In glandular enlargements and malignant growths, its use is more beneficial than most other stimulants, in bronchocele and other affections of the throat, and thyroid glands iodine is considered invaluable. As the Dentist is not expected to treat such diseases the further consideration of the agent in this connection will be discontinued. It has only been thus spoken of to show its efficacy in such cases.

Iodine is less used by the Dentist than the tincture ; as an internal remedy it is seldom used, the iodide of potassa being considered far superior. Iodine may be useful in the local treatment of chronic inflammation or induration of the salivary glands, in dental periostitis, in alveolar abscess, in some morbid states of the antrum, in thickening of the mucus membrane, in tumors of the mouth, and in absorption of the gums and alveolar processes. The officinal tincture will answer very well for periostitis, thickening of the membrane, and sometimes for abscess. For chronic catarrh, or inflamation of the lining membrane of the antrum, the compound tincture very much diluted is a good injection. For destroying the sac in alveolar abscess, a solution of iodine and creosote is a sovereign agent, and when not too concentrated, the same is an admirable application to the margins of the gums and alveolar processes, after the removal of all irritating and dead substances ; but care must be taken that it is not applied too frequently. It is an escharotic, and as a general rule it is well to let the slough separate before a second application. To use externally I prefer a colorless solution of iodine, prepared by combining equal quantities of compound tincture of iodine and pure aqua ammonia. As combination takes place, the mixture becomes transparent and will not then color the skin. I do not think of any condition which will require the Dentist to prescribe iodine internally. When its constitutional action is indicated as in scrofulous or syphilitic diseases of the mouth more benefit will be derived from the use of iodide of potassium.[1]

### IODIDE OF POTASSIUM.

Omitting its history, we will pass immediately to its physiological effects and uses.

"Locally, this salt is an irritant, but is not near so energetic in its action as free iodine. On this account it may be given internally, in larger doses and for a longer period, than iodine. Indeed, iodine can be introduced into the system much faster by the use of the iodide, than when given uncombined. A solution of albumen, fibrin, or gelatin, is not obviously changed by the addition of this salt, and as these are the most abundant organic constituents of the body, we may infer that the chemical action of iodide on the living tissue is but slight. To obtain a clear view of the action of this salt, as a remedial agent, it is necessary to bear in mind its peculiar properties. It is very soluble, and is, therefore, readily absorbed. It passes rapidly into the circulation, and may be detected in all the tissues and secretions. It is composed of two elements, both of which are characterized by strong affinities for other substances, and for some of them stronger than that by which they are held together.

If the salt is decomposed, the potassium takes oxygen and becomes potash, which is a general solvent of the animal tissues. At the same time the iodine is set free, and is thus able to exert its affinities. And as all chemical agents are peculiarly active in the nascent condition, the iodine and potash are both more energetic than if carried in their free state, to the point of action. Each one, as it were, holds the other quiet till the proper point is reached, and then lets it go, to accomplish its work. Each element by neutralizing the other prevents its local, *irritant* action, and each is liberated, atom by atom, in obedience to stronger affinities, each

1. Watt's Dental Materia Medica.

particle being promptly saturated, by the gratification of the affinity, which liberates it. And this explains how it is that such large doses of this salt, can be taken for a long time, without local, or constitutional disturbance. The affinities of iodine and potassa are sufficient to account for all the phenomena, observed in the remedial action of the salt. Highly soluble compounds, are the natural results, and these are naturally carried out by the various excretories. Let us suppose that the iodide is administered for the arrest or removal of morbid growths, or to relieve tertiary syphilis. The latter is often spoken of as a disease of the bones. But does that expression convey the whole truth? Is there not a disease of the formative fluids from which bony tissue is deposited? Now, if these morbid particles are arrested, by the affinities of the elements of the salt, held in solution, and carried out through the various secretions, it is evident they will not further build up the morbid development. And as the particles of morbid structure, like those of normal tissues, perform their functions and pass away, unless new ones are furnished in their places, the abnormal solid is carried off little by little. The diseased growth is literally starved to death, and carried out by the scavengers of the system. It is this action of the remedy which has induced some writers to call it a resolvent, or liquefacient. In some of the above remarks, I have sacrificed technicality, to a desire to be understood by beginners, and those whose opportunities have not been such as they desired. They are not written for the critic, though of course, he may use his pleasure in regard to them. It should be given in solution, and usually, immediately after eating. It may be taken in sweetened water, or almost any way the patient may fancy. The average dose is from 4 to 10 grains. Many use much larger doses, but I have not found it best to do so. For an adult, I frequently prescribe a solution of a drachm of the salt to an ounce of water, and direct the patient to take a teaspoonful three times a day."

1. Watt's Dental Materia Medica.

### BROMINE.

Bromine is a volatile liquid of a dark red color, when viewed in substance. Its taste is very caustic, and its smell very disagreeable, somewhat resembling chlorine. It evaporates rapidly, and is sparingly soluble in water, more so in alcohol, and still more in ether. It is valuable for its bleaching properties, and may be used for bleaching teeth, not so well, however, as chlorine, or chlorate of zinc. Bromine is intermediate in its effects between iodine and chlorine. It stimulates the sympathetic system, promotes absorption, and is supposed to be more energetic than iodine or bromide of mercury. It is recommended where iodine has been tried, and does not act with sufficient energy, or has lost its efficacy by habit. I am of opinion that bromine, like iodine, is not as efficacious as bromide of potassium. In case the patient has any syphilitic taint, bromine and bromide of potassium may advantageously be used. They may be used constitutionally or locally, either or both if thought best, for local treatment, make a saturated solution of bromide of potassium, then add 40 drops of bromine to each ounce of the solution, and apply to the effected part, always cleansing the part well before making the application. Apply this remedy in the same manner as creosote. For internal treatment take of bromide of potassium 1℈, distilled water 1℥, misce, and add bromine 1℈. Take a teaspoonful three times a day, one hour before or after each meal. This treatment is only to be used in cases of syphilitic taint.

When local treatment is applied through the canal, it should first be cleansed of all impurities, such as nerve membrane, or any foreign substance contained therein, and the root opened freely to allow a free use of the injection. In case the discharge be fetid, a solution of chloride of sodium should be injected into the cavity, to correct this condition. After this, an injection of any of the above agents, may be used to break down the sac. The directions for the use of which see CREOSOTE.

Here let me remark, that the young and inexperienced may be easily deceived in their cure. Either of the local remedies used will soon impart a very healthy appearance, and often cause the external opening to heal almost immediately, causing the operator to think he has cured the disease, when really he has hardly checked it. It is frequently the case that the therapeutic treatment alone will not affect a cure, but surgical aid is required. In the treatment of abscess in the inferior Maxilla, there are serious difficulties, which are not met with in the superior. One is, the situation being at the bottom, instead of at the top of the socket, the secretions rest on the diseased parts, while in the superior it is drained off. The presence of this matter is a serious obstacle in the treatment of abscess unless it can be drained off and kept free. Again, the size and shape of the jaw, is such that an opening through the gums can not be well made. Therapeutic treatment, in cases of this kind, is not very efficient unless it be vigorous. The treatment of abscess in the inferior jaw is not generally so successful as that of the superior.

### SURGICAL TREATMENT.

The surgical treatment of alveolar abscess is very short and simple. That most commonly resorted to, viz: extraction, is generally successful. There are cases, however, in which it is desirable to avoid the extraction of the tooth, but, there are a great many teeth thus affected that are utterly useless and should be removed; for the irritation which they cause to the surrounding parts, to say nothing of the abscess, is sufficient cause for their removal. Old roots of teeth, and teeth that have lost their antagonists are nearly always a source of irritation, and when that is the case they should be removed. It sometimes happens that an abscess at the root of a tooth, will burrow into the alveolar process, making the cavity containing the abscess larger a short distance beyond the root than at the socket. In such a case the sac will nearly always be retained in the alveolus after the tooth or root is removed; the abscess now acts independent of the cause which produced it, and the extraction of the tooth will rarely effect a cure, and an operation for the removal of the abscess will be necessary.

When the tooth is a valuable one and should be retained, either for ornament or service, and therapeutic treatment does not accomplish the desired result, it may be aided by the trephine, drill or chisel; with either of these instruments an opening may be made through the alveolus opposite the point of the root of the affected tooth, then, with a suitable instrument cut away or separate the sac from the root.

In some cases of alveolar abscess the alveolus is largely affected, and in such a diseased condition that it will not heal without first cleansing the parts; this may be done by chiseling or scraping off all dead particles of bone in and about the cavity, for as long as anything of the kind remains, the chances of success are greatly diminished. Care should be taken in all cases, that unnecessary pain is not inflicted. I have met and treated cases of abscess, where the outer wall of the alveolus was so largely affected as to present a honey-comb appearance; the only speedy and successful treatment in such a case, would be a breaking down of the diseased wall, and the removal of every particle of diseased bone. Again, in addition to the above, the root of the tooth may be in such a diseased condition, that the removal of the diseased portion of its substance will be necessary to a cure. All diseased bone and tooth substance being removed, a proper therapeutic treatment, and a vigorous constitution, will soon affect a cure. Compresses are necessary to stay the tide of nutritious food to the abscess, together with all other means that assist in the abatement of the disease.

To sum up the whole treatment, in a few words, the forceps and the chisel are the most effective instruments, and will generally be found successful. In the incipient stage of an abscess, if it be at a point where the outer wall of the alveolus is thin, and easily cut through, the knife may be used to advantage, by cutting through the process to the abscess, which will greatly facilitate the escape of the pus, and in this way a cure is sometimes effected. Scarrifying the gums, and thoroughly opening down to the sac with the knife is often successful. These are the surgical means usually employed, and are so plain that the time, and manner need not be misunderstood.

### EVIL RESULTS.

We wish now to call attention to some of the evil results of alveolar abscess. I desire to call attention first to children's teeth. In these the greatest evil and most to be dreaded is necrosis which may take place, and extend to the sockets of the permanent teeth, causing exfoliation of their walls, as well as those of the temporary teeth. This is of the utmost importance, as by the destruction of the alveolus the permanent teeth are also very often lost. There have been several cases where the disease occasioned by an abscess caused exfoliation of the sockets of two or three teeth. It is frequently the case where the first or second superior molars are effected, their roots being situated immediately beneath the floor of the antrum, and as the roots very closely approximate, and sometimes even penetrate it, an abscess of these teeth often produces a disease in this cavity, that is very troublesome, and may result in Hydatides of the antrum. They are often very hard to cure, and in some cases are never cured. This is a very serious form of disease, superinduced by abscess. We find about as extensive, and alarming troubles arising from an abscess of the inferior third molars, as in any other of the mouth. Some of the reasons for this are, first, the difficulty of diagnosing the disease. We find physicians, as a general rule, liable to be misled, and even Dentists are not always free from being deceived, in consequence of the opening for the escape of the pus, being at a considerable distance from the seat of the disease, the patient is often treated for a different disease entirely. Such has been, is now, and will continue to be the case, for those who are called in such cases treat their patients for months, and even years, without knowing what is the real cause of the disease, while, in the meantime, the abscess is still progressing, and as the tooth is situated so closely to the fauces, and soft parts, they will soon become largely inflamed, and if the tooth is very much decayed, the evil is increased by the amount of irritation produced by the ragged edges of the tooth, and thus extending to the lungs, may, so seriously effect the lungs as to finally produce *Phthisis Pulmonalis.* Again, the amount of swelling and inflammation in the mastoid muscles may be such as to render them useless, for the time being, and if the disease continues for any considerable length of time the muscles become ridged, and finally the jaws can not be used with that freedom they should be.

Exfoliation of the alveolus is another of the evils of alveolar abscess. This was mentioned above, but only in connection with children's teeth. I now recur to it mainly for the purpose of giving it a more extended notice, and relate a case or two to show how far this disease will be carried when all things are favorable. When the inflammation is extreme or very great it may produce necrosis, and exfoliation. In an old work published by "Fox & Harris on the Human Teeth," two or three cases are recorded, in two of which, three teeth, and in the third, all of the anterior teeth were lost by the suppurative process, produced by an abscess.

It is unnecessary to mention other cases to prove the evils of the disease in question, any one will admit that those already mentioned are quite sufficient to show the importance of timely attention. With regard to the treatment of abscess there is yet very much to be learned; in fact very little as compared to what is required is yet thoroughly understood. Yet, enough is known to prove conclusively that very many cases may and can be cured. But the progressive practitioner will not rest contented with what is known on the subject.

